# Supplementary material for: Accurate Determination of the Degree of Deacetylation of Chitosan Using UPLC–MS/MS
Source: Int J Mol Sci. 2022 Aug 8;23(15):8810. doi: 10.3390/ijms23158810 (PMC9369293; doi:10.3390/ijms23158810)
Supplement: Supplementary file 1 [file ijms-23-08810-s001.zip › ijms-1809299-supplementary.pdf]

SUPPLEMENTARY MATERIAL

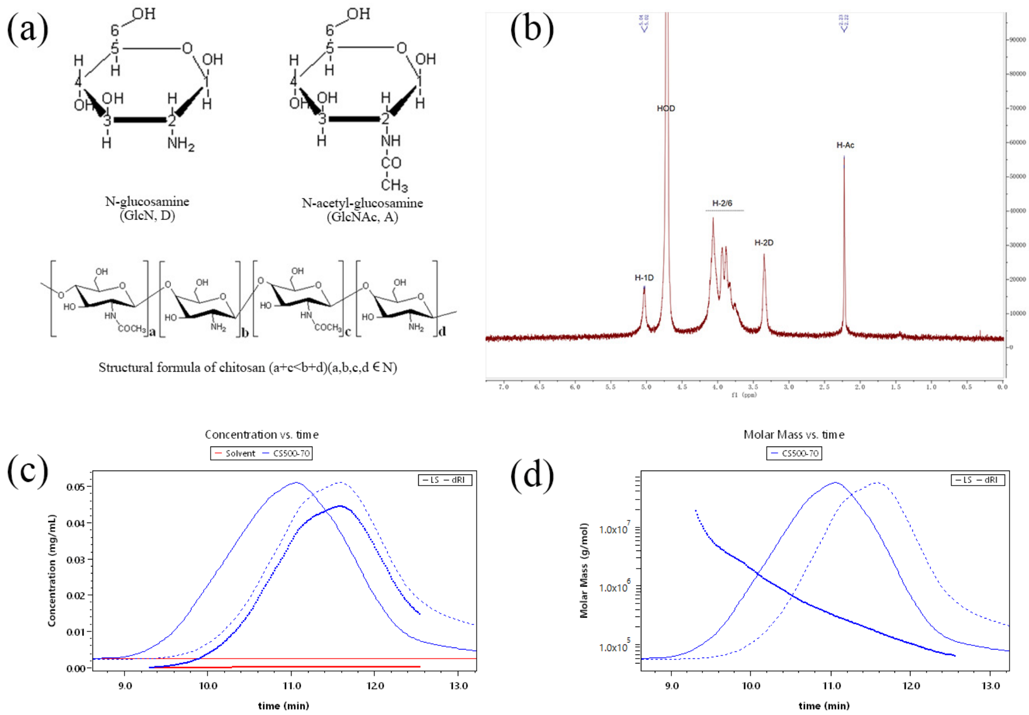

Figure S1. (a) The structural formula of N-glucosamine (GlcN, D) units; N-acetyl-glucosamine (GlcNAc, A); and chitosan (CS). (b) The proton nuclear magnetic resonance (^{1}H NMR) spectrum of CS500-70; (c) size exclusion chromatography-multiangle light scattering (SEC-MALS) chromatograms of the solvent (red line) and CS500-70 (blue line); (d) Molar mass (blue heavy solid line) as a function of elution time, LS responses at 90.0° (blue thin solid line) and refractive index detector (RID) responses (blue thin dashed line) for CS500-70.

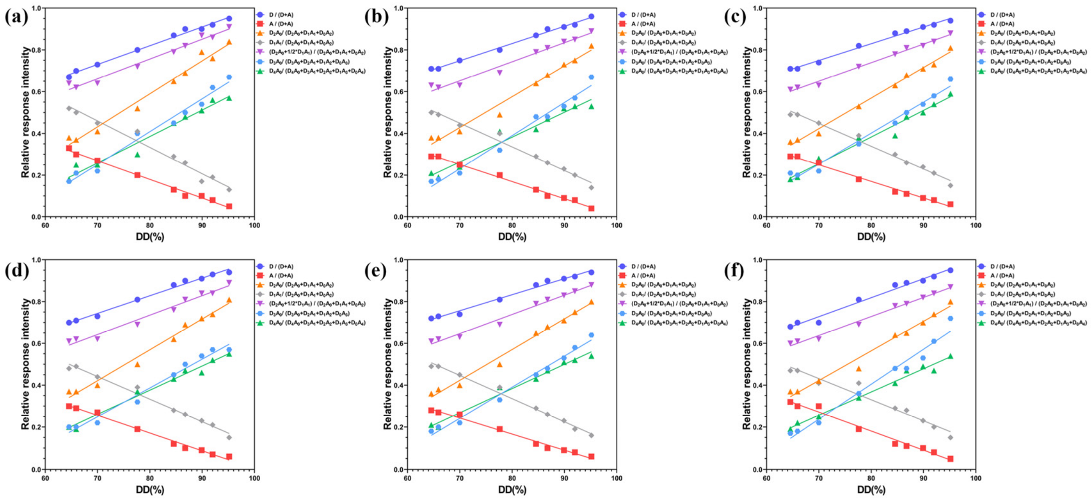

Figure S2. Linear relationships between the relative response intensity (RRI) of characteristic ion pairs of chitosan and the DD by detection using different mass spectrometry parameters. (a–b) different capillary voltages, 1000 and 4000 V; (c) temperature, 400 °C; (d–f) desolvation gas flow, 700 L/Hr; cone gas flow, 200 L/Hr; nebulizer gas flow, 5.5 Bar.

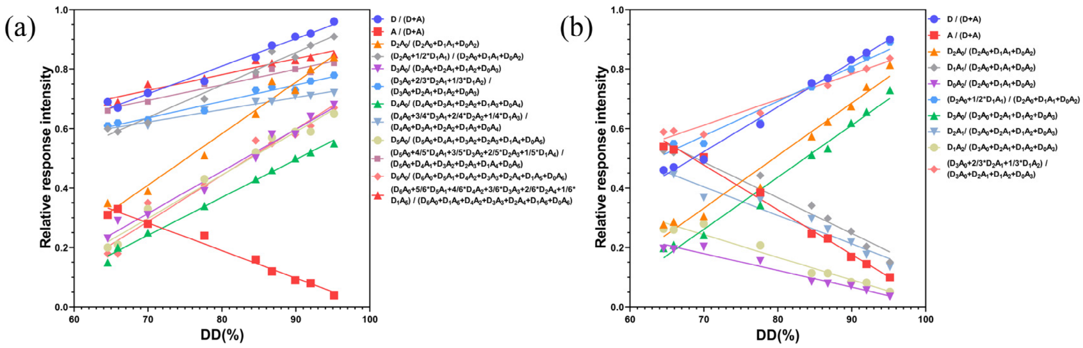

Figure S3. Linear relationships between the relative response intensity (RRI) of the characteristic ion pairs of chitosan and the DD through detection on SCIEX Triple Quad™ 6500plus (a) and API4000 mass spectrometers (b).

Table S1. The relative errors (RE) between the degree of deacetylation (DD) of chitosan calculated using the established standard curve and that measured by ^{1}H NMR.

Sample	^{1}H NMR DD(%)	D / (D+A)		A / (D+A)		$D_2A_0 / (D_2A_0+D_1A_1+D_0A_2)$		$(D_2A_0+1/2*D_1A_1) / (D_2A_0+D_1A_1+D_0A_2)$		$D_4A_0 / (D_4A_0+D_3A_1+D_2A_2+D_1A_3+D_0A_4)$		$D_5A_0 / (D_5A_0+D_4A_1+D_3A_2+D_2A_3+D_1A_4+D_0A_5)$		$(D_5A_0+4/5*D_4A_1+3/5*D_3A_2+2/5*D_2A_3+1/5*D_1A_4) / (D_5A_0+D_4A_1+D_3A_2+D_2A_3+D_1A_4+D_0A_5)$		$D_6A_0 / (D_6A_0+D_5A_1+D_4A_2+D_3A_3+D_2A_4+D_1A_5+D_0A_6)$	
		Calculated DD(%)	RE(%)	Calculated DD(%)	RE(%)	Calculated DD(%)	RE(%)	Calculated DD(%)	RE(%)	Calculated DD(%)	RE(%)	Calculated DD(%)	RE(%)	Calculated DD(%)	RE(%)	Calculated DD(%)	RE(%)
CS 1100-65	64.54	64.80	0.40%	64.80	0.40%	65.27	1.12%	65.23	1.06%	65.32	1.21%	64.30	-0.38%	64.79	0.38%	65.31	1.19%
CS 600-66	65.91	64.72	-1.80%	64.72	-1.80%	66.12	0.32%	65.97	0.10%	66.20	0.44%	65.72	-0.28%	65.90	-0.01%	65.02	-1.35%
CS 500-70	69.97	70.74	1.10%	70.74	1.10%	69.99	0.03%	69.74	-0.33%	69.68	-0.42%	71.01	1.48%	69.41	-0.80%	69.75	-0.31%
CS 500-78	77.62	77.38	-0.31%	77.38	-0.31%	76.50	-1.45%	76.75	-1.12%	76.12	-1.93%	76.63	-1.28%	77.02	-0.78%	78.29	0.86%
CS 400-85	84.57	85.81	1.47%	85.81	1.47%	83.08	-1.75%	83.59	-1.16%	84.48	-0.10%	84.45	-0.14%	85.49	1.09%	85.34	0.91%
CS 200-87	86.76	87.23	0.55%	87.23	0.55%	87.76	1.16%	88.14	1.59%	87.63	1.01%	87.76	1.15%	88.37	1.85%	85.28	-1.70%
CS 600-90	89.90	88.88	-1.14%	88.88	-1.14%	89.29	-0.68%	89.21	-0.77%	89.03	-0.97%	89.11	-0.89%	88.88	-1.13%	88.77	-1.26%
CS 300-92	91.99	92.01	0.02%	92.01	0.02%	92.41	0.45%	92.21	0.24%	91.77	-0.24%	92.43	0.48%	91.79	-0.22%	94.02	2.21%
CS 300-95	95.17	94.86	-0.32%	94.86	-0.32%	96.01	0.88%	95.59	0.44%	96.18	1.06%	95.03	-0.14%	94.78	-0.41%	94.65	-0.55%

Table S2. The mean and standard deviation of the degree of deacetylation (DD, %) (mean ± SD, n= 6) detected using UPLC-MS/MS of the relative response intensity (RRI) of D / (D+A), A / (D+A), D_2A_0 / ($D_2A_0+D_1A_1+D_0A_2$), ($D_2A_0+1/2*D_1A_1$) / ($D_2A_0+D_1A_1+D_0A_2$), D_4A_0 / ($D_4A_0+D_3A_1+D_2A_2+D_1A_3+D_0A_4$), D_5A_0 / ($D_5A_0+D_4A_1+D_3A_2+D_2A_3+D_1A_4+D_0A_5$), ($D_5A_0+4/5*D_4A_1+3/5*D_3A_2+2/5*D_2A_3+1/5*D_1A_4$) / ($D_5A_0+D_4A_1+D_3A_2+D_2A_3+D_1A_4+D_0A_5$) and D_6A_0 / ($D_6A_0+D_5A_1+D_4A_2+D_3A_3+D_2A_4+D_1A_5+D_0A_6$) for 9 chitosans.

Sample	^{1}H NMR DD(%)	UPLC-MS/MS DD(%)							
		D / (D+A)	A / (D+A)	$D_2A_0 / (D_2A_0+D_1A_1+D_0A_2)$	$(D_2A_0+1/2*D_1A_1) / (D_2A_0+D_1A_1+D_0A_2)$	$D_4A_0 / (D_4A_0+D_3A_1+D_2A_2+D_1A_3+D_0A_4)$	$D_5A_0 / (D_5A_0+D_4A_1+D_3A_2+D_2A_3+D_1A_4+D_0A_5)$	$(D_5A_0+4/5*D_4A_1+3/5*D_3A_2+2/5*D_2A_3+1/5*D_1A_4) / (D_5A_0+D_4A_1+D_3A_2+D_2A_3+D_1A_4+D_0A_5)$	$D_6A_0 / (D_6A_0+D_5A_1+D_4A_2+D_3A_3+D_2A_4+D_1A_5+D_0A_6)$
CS 1100-65	64.54	64.26±0.78	64.26±0.78	65.43±0.57	65.52±0.51	64.53±0.51	64.59±0.65	65.47±0.82	63.98±0.90
CS 600-66	65.91	66.27±1.15	66.27±1.15	66.57±0.54	66.48±0.68	65.59±0.63	64.96±0.64	65.50±0.64	66.02±0.60
CS 500-70	69.97	69.22±1.01	69.22±1.01	69.49±0.38	69.10±0.41	70.21±0.72	71.62±0.61	69.32±0.67	70.30±0.55
CS 500-78	77.62	77.59±0.31	77.59±0.31	75.76±0.67	75.91±0.75	77.26±0.73	76.34±0.47	76.34±0.69	78.05±1.05
CS 400-85	84.57	85.75±0.50	85.75±0.50	84.18±0.69	84.71±0.76	85.25±0.92	84.50±1.18	85.75±0.74	84.68±0.97
CS 200-87	86.76	87.72±0.51	87.72±0.51	87.51±0.37	87.71±0.39	87.37±0.31	88.33±0.64	88.67±0.57	87.33±1.64
CS 600-90	89.90	89.37±0.61	89.37±0.61	88.99±0.73	89.13±0.59	89.67±0.63	88.91±0.31	88.93±0.40	87.79±1.39
CS 300-92	91.99	91.85±0.17	91.85±0.17	92.29±0.43	92.01±0.35	91.65±0.38	92.00±0.35	91.72±0.31	93.00±1.50
CS 300-95	95.17	94.38±0.40	94.38±0.40	96.21±0.28	95.87±0.28	94.90±0.87	95.18±0.48	94.74±0.48	95.27±0.95
